# Thickness-dependent conductance in Sb_2_SeTe_2_ topological insulator nanosheets

**DOI:** 10.1038/s41598-017-02102-7

**Published:** 2017-05-15

**Authors:** Shiu-Ming Huang, You-Jhih Yan, Shih-Hsun Yu, Mitch Chou

**Affiliations:** 1 Department of Physics, National Sun Yat-Sen University, Kaohsiung, 80424 Taiwan; 2 Department of Materials and Optoelectronic Science, National Sun Yat-Sen University, Kaohsiung, 80424 Taiwan; 3 Taiwan Consortium of Emergent Crystalline Materials, TCECM, National Sun Yat-Sen University, Kaohsiung, 80424 Taiwan

## Abstract

The conductivity increases as thickness decreases in a series of Sb_2_SeTe_2_ topological insulator nanosheets with thickness ranging from 80 to 200 nm, where the sheet conductance is proportional to the thickness. The corresponding sheet conductance of the surface state is 8.7 e^2^/h which is consistent with the values extracted from the temperature dependent Shubnikov-de Haas oscillations at high magnetic fields. The extracted Fermi momentum is the same as the results from the ARPES value, and the Berry phase is *π*. These support that the thickness dependent sheet conductance originates from the combination of the surface state and the bulk state.

## Introduction

Three-dimensional topological insulators possess a linear dispersion gapless surface state that is protected by time-reversal symmetry^1, 2^. The topological surface state consists of spin filtered Dirac fermions. This spin helical texture of the topological surface state has attracted a great deal of attention due to its possible electric and spin-related applications^3–18^. The carrier transport characteristics of this kind of topological insulators are contributed by both the surface state and the bulk state. The carrier trasnport of the surface state is on the species’ surface; thus, the total carrier transport characteristic would be directly related to the thickness. To identify the transport characteristics, as well as the quantum Shubnikov-de Haas (SdH) oscillation at high magnetic fields and low temperatures, the thickness dependent conductance is an appropriate platform to detect the transport characteristics of the topological insulator surface state in ambient conditions.

However, the quick surface oxidation strongly influences the transport properties in thin topological insulator films, and this effect is more serious in thinner films. On the other hand, to grow a large-scale topological insulator film with high homogeneity is a challenge, as the film inhomogeneity leads to sample-dependent deviation. Due to these effects, there are few studies on the thickness-dependent transport characteristics of topological insulators^19–23^. The previous works on the thickness-dependent transport properties reveal that the data fluctuation is large and the sheet conductance might reach 1 order of scale deviation. Furthermore, the reported thickness-dependent transport properties are not consistent^19–23^. It is believed that this deviation might come from the unavoidable and uncontrollable surface oxidation during the fabrication and experimental processes, and/or the inhomogeneous films^24^. To overcome this obstacle, a surface oxidation resistant and highly uniform topological insulator is a proper system to clarify this problem. Our previous work supports that the Sb_2_SeTe_2_ topological insulator is tolerant to the surface oxidation, and the crystal uniformity can reach the cm-scale^25–27^. It is therefore a suitable material to investigate the thickness-dependent carrier transport characteristics of the surface state.

In this paper, the thickness-dependent conductance is performed in the Sb_2_SeTe_2_ and reveals that the sheet conductance is proportional to the thickness. The determined sheet conductance of the surface state is consistent with the extracted values from the quantum SdH oscillation from the surface state of the Sb_2_SeTe_2_, and the carrier transport characteristics of the topological surface state were systematically determined.

## Experimental Methods

The single crystals of Sb_2_SeTe_2_ were grown with a homemade resistance-heated floating zone furnace (RHFZ). The starting raw materials of Sb_2_SeTe_2_ were mixed according to the stoichiometric ratio. At first, the stoichiometric mixtures of high purity elements Sb (99.995%), Se (99.995%) and Te (99.995%) were melted at 700∼800 °C for 20 h and then slowly cooled to room temperature in an evacuated quartz glass tube. The material was used as a feeding rod for the following RHFZ experiment. Our previous work supports that extremely high crystal uniformity in topological insulator crystals can be obtained through the RHFZ method^25, 27^. After growth, the crystals were then furnace cooled to room temperature. The as-grown crystals were cleaved along the basal plane, with a silvery shiny mirror-like surface, and then prepared for the further experiments. The EDS results support that the Sb:Se:Te = 2:1:2 and the XRD spectrum is consistent with the database of Sb_2_SeTe_2_^25, 28^.

**Figure 1:**
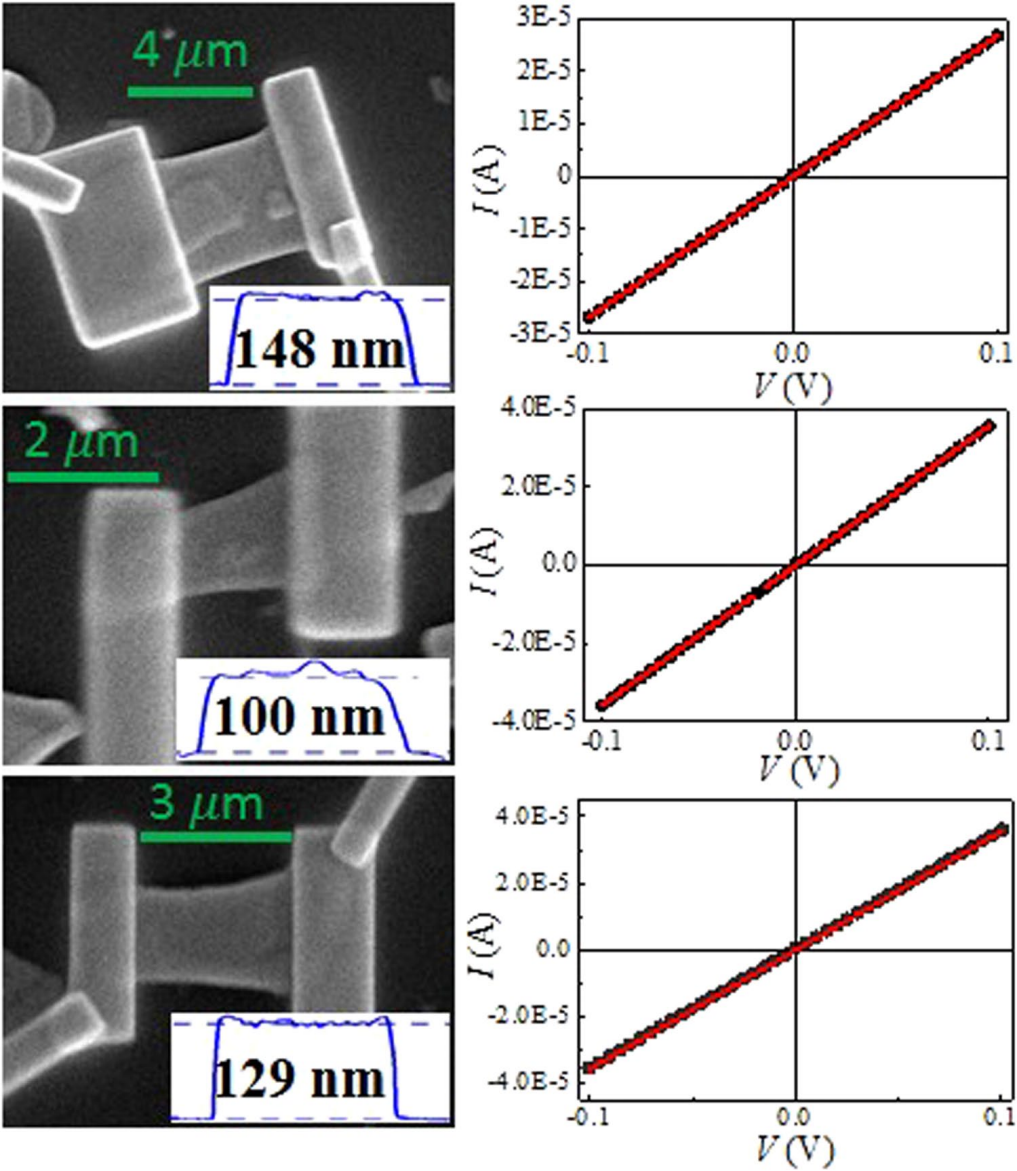
The SEM pictures of the Sb_2_SeTe_2_ nanosheets of different thicknesses. The current-voltage shows the linear relation that supports the ohmic contacts of our samples. The conductance is determined by the slope of the current-voltage curve.

The cleaved Sb_2_SeTe_2_ single crystals were dispersed on the insulating SiO_2_/*n*-Si templates^29^. The ohmic Pt contacts were fabricated using Focused-ion beam deposition. The thickness of the Pt contacts was roughly sub-micro meter. Figure 1 shows the SEM picture of the Sb_2_SeTe_2_ nanosheets. It shows the linear current-voltage relation that indicates the ohmic contact between the Pt contacts and the Sb_2_SeTe_2_ nanosheets. The conductance is determined by the current-voltage slope at room temperature. Another six-probe sample was fabricated to performed the SdH oscillations at low temperatures. The sample is 4-mm in width, 6-mm in length and 0.1-mm in thickness. Magnetotransport measurements were performed using the standard six-probe technique in a commercial apparatus (Quantum Design PPMS) with a magnetic field of up to 9 T. The magnetic field was applied perpendicular to the large cleaved surface.

## Results and Discussion

**Figure 2:**
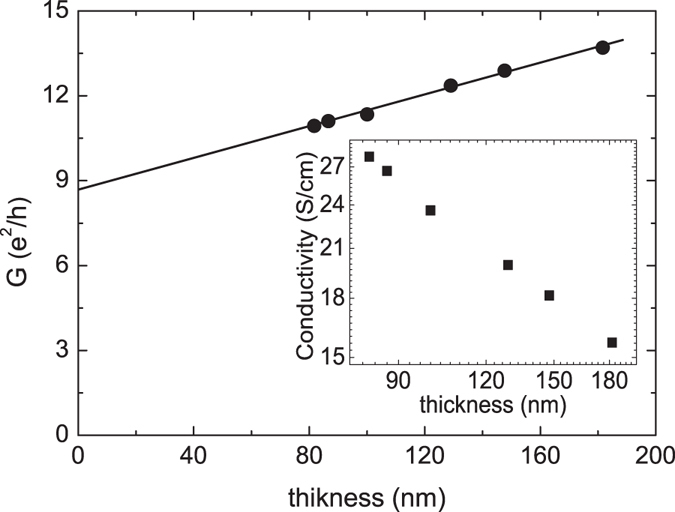
The inset shows the conductivity as a function of thickness in a log-log plot. The conductance decreases as the thickness increases. The sheet conductance is proportional to the thickness. The y-intercept is 8.7 (e^2^/h) which is the sheet conductance of the surface state. The slope is 2.76 × 10^−2^ (e^2^/h) nm^−1^ which is the conductivity of the bulk state.

The inset of Fig. 2 shows the log-log plot of the conductivity as a function of the thickness, and reveals the thickness-dependent conductivity for samples with the thickness ranging from 80 to 200 nm. It differs from the conventional materials for which the conductivity is independent of the thickness. The measured conductivity decreases as the thickness increases, which is similar to the reported behavior in the two-dimensional transition metal dichalcogenides (TMDs)^29^, and is ascribed to the transport characteristic of the layer carriers. The conductivity, *σ*, is inversely proportional to the thickness, *σ* ∝ *t* ^−1^, in a system with layer carriers. A dependence of the inverse power-law, *σ* ∝ *t* ^−*α*^, was used for fitting our data. The results show that *α* is about 0.72, which obviously deviates from 1. On the other hand, it is known that the sheet conductance, *G*, which is defined as *σt*, is a characteristic of layer transport carriers. The product of *σt* is thickness-independent in a layer transport system. Figure 2 shows the sheet conductance as a function of the thickness. The sheet conductance is proportional to the thickness. This is different from the reported result that the sheet conductance is independent of the thickness in 2D TMDs^29^. These results support that other sources contribute to the measured conductivity in addition to the layer transport carriers^21^. We propose that these characteristics originate from the peculiar band structure of the topological insulator, and the observed thickness-dependent sheet conductance originates from the combination of the surface state and the bulk state. The total sheet conductance *G* of a topological insulator nanosheet with a thickness *t* could be expressed as:

1 $$G={G}_{s}+{G}_{b}={G}_{s}+{\sigma }_{b}t$$

where *G*_*s*_ is the sheet conductance of the surface state, *G*_*b*_ is the sheet conductance of the bulk state, and *σ*_*b*_ is the conductivity of the bulk state. As shown in Fig. 2, the measured sheet conductance is proportional to the thickness. The extracted *G*_*s*_ = 8.7 (e^2^/h) and *σ*_*b*_ = 2.76 × 10^−2^ (e^2^/h) nm ^−1^. *σ*_*b*_ is larger than the reported value in the Bi_1.5_ Sb_0.5_ Te_1.7_ Se_1.3_ topological insulator nanoflake by a factor of 5^21^.

**Figure 3:**
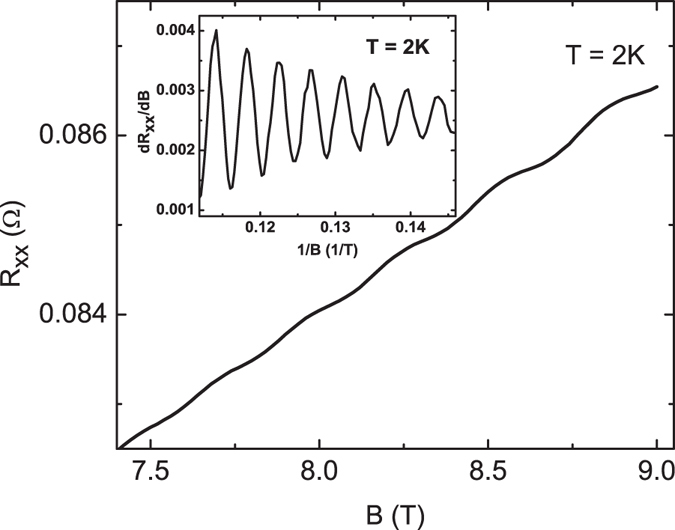
The magnetic field dependence of the resistance. The oscillation behavior is observed at high magnetic fields. The inset shows the plot of *dR*_*xx*_/*dB* as a function of *B*, and shows an SdH oscillation.

**Figure 4:**
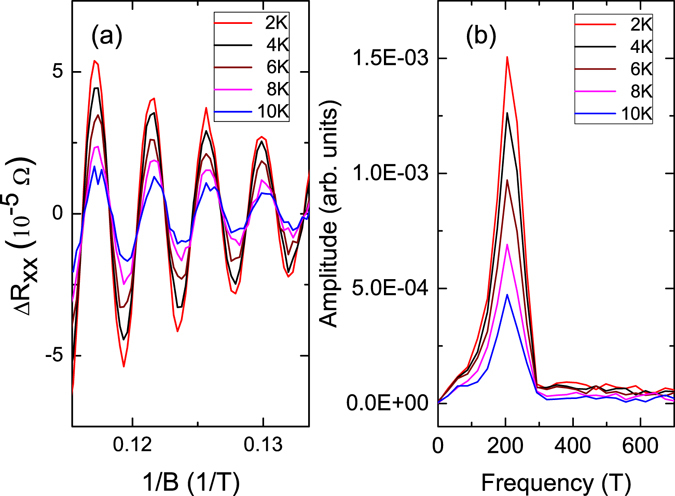
(**a**) SdH oscillation as a function of 1/*B* at several temperatures of Sb_2_SeTe_2_. The oscillation periods are the same and the peak height decreases as the temperature increases. (**b**) The amplitude of the Fourier transform of the Δ*R*_*xx*_ as a function of *B*. The peaks, *F* = 205 T, are the same for the measured temperatures.

To further confirm that the thickness-dependent sheet conductance originates from the characteristics of the surface state, the surface state dominated quantum Shubnikov-de Haas (SdH) oscillation is performed. Figure 3 shows the magnetoresistance *R*_*xx*_ at 2 K, and the oscillation behavior is observed at high magnetic fields. As shown in the upper inset of Fig. 3, *dR*_*xx*_/*dB* is plotted as a function of inverse magnetic fields, and a clear periodic oscillation is observed. The Δ*R*_*xx*_(*B*) is plotted as a function of the 1/*B* for several temperatures. (The Δ*R*_*xx*_(*B*) is the measured *R*_*xx*_ substrates smooth polynomial background function). As shown in Fig. 4(a), the oscillation periods are the same for all measured temperatures. The oscillation amplitude decreases inversely as the magnetic field increases, and decreases as the temperature increases. This confirms that the observed oscillation is SdH originating from the surface state of Sb_2_SeTe_2_. The SdH oscillation frequencies are extracted from the Fourier transform, and as shown in Fig. 4(b), a major peak at a frequency of *F* ≈ 205 T is observed for all measured temperatures. This oscillation frequency is consistent with the reported value from the surface state of the Sb_2_SeTe_2_ topological insulator^30^.

The SdH oscillation frequency, *F*, is directly related to the Fermi-level cross section via the Onsager relation $F=(\frac{\hslash c}{2e}){k}_{F}^{2}$, where *k*_*F*_ is the Fermi vector, *e* is the electron charge and $\hslash$ is the Planck constant. The observed SdH oscillation frequency, *F* = 205 T, leads to *k*_*F*_ = 7.8 × 10^6^ cm^−1^, which is consistent with the value of *k*_*F*_ = 7.3 × 10^6^ cm^−1^ extracted from ARPES from the different batches of the same crystal^26^. It is reported that the *k*_*F*_ extracted from ARPES and SdH oscillation are different because of the sample inhomogeneity^25, 27^. Our ARPES and SdH oscillation show consistent results that support the high homogeneity of our samples^25, 26^. The *k*_*F*_ deduces the surface density of state through the relation *n*_*s*_ = *k*_*F*_^2^/4*π* = 4.95 × 10^12^ cm^−2^ for a circular Fermi surface.

Following the Lifshitz-Kosevich (LK) theory, the temperature dependence of the amplitude of the SdH oscillation is expressed as

2 $${\rm{\Delta }}{R}_{xx}(T,B)\propto \frac{\mathrm{((2}{\pi }^{2}{k}_{B}T)/{\rm{\Delta }}{E}_{N}(B)){e}^{-\mathrm{(2}{\pi }^{2}{k}_{B}{T}_{D})/{\rm{\Delta }}{E}_{N}(B)}}{\sin \,h\mathrm{((2}{\pi }^{2}{k}_{B}T)/{\rm{\Delta }}{E}_{N}(B))},$$

**Figure 5:**
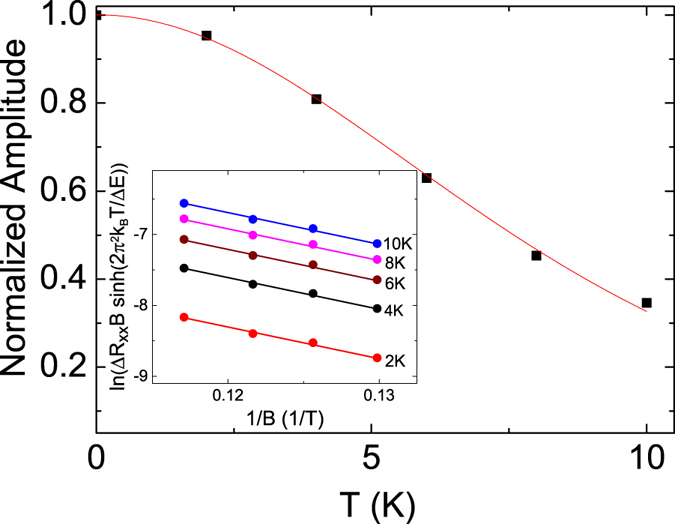
The temperature dependence of the normalized amplitude of SdH oscillation is at 8.8 T. The result corresponds well with the Lifshitz-Kosevich theory. The inset shows the oscillation amplitude as a function of inverse magnetic fields at several temperatures. The Dingle temperature is 17 K.

The Landau level spacing ${\rm{\Delta }}{E}_{N}(B)=\hslash eB/{m}_{cyc}$. Figure 5 shows the normalized oscillation amplitude at *B* = 8.8 T as a function of temperature. The Δ*E*_*N*_ can be determined for different field values from Δ*R*_*xx*_(*T*). The linear fitting yields a cyclotron mass *m*_*cyc*_ = 0.17*m*_0_ where *m*_0_ is the free electron mass^31–33^. Following the linear dispersion relation, ${v}_{F}=\hslash {k}_{F}/{m}_{cyc}$, the Fermi velocity of the carriers in the surface state is determined as *v*_*F*_ = 5.26 × 10^7^ cm/s.

To extract the Dingle temperature, as shown in the inset of Fig. 5, the oscillation amplitude as a function of the inverse magnetic field at several temperatures is plotted. The Dingle temperature of *T*_*D*_ = 17 K is determined from the slope in the semilog plot of Δ*R*_*xx*_ *B*sin*h*((2*π* ^2^ *k*_*B*_ *T*)/Δ*E*_*N*_(*B*)) versus 1/*B*. The corresponding surface carrier lifetime, *τ* = 7.1 × 10^−14^ s, is determined through the relation, ${T}_{D}=\hslash /(2\pi {k}_{B}\tau )$. The corresponding mean free path, ${l}_{s}={v}_{f}\tau =37.3$ nm, and the carrier mobility, ${\mu }_{s}=(e{l}_{s})/(\hslash {k}_{F})=718$ cm^2^/*Vs*, of the surface carrier are determined. This mobility is small compared to the other observed values in the literature^33–35^. The surface state of topological insulators comes from the strong spin-orbit interaction. The Bi atom is heavier than the Sb atom and that might be a possible reason that the observed carrier mobility in our Sb_2_SeTe_2_ topological insulator is lower than the reported values in Bi-base topological insulators. On the other hand, it is widely known that the different growth methods lead to different characteristics. Our Sb_2_SeTe_2_ single crystal was grown through the homemade heat-melting furnace; that is different from most of the reported method such as MBE, pulse deposed method and Bridgeman method. The mechanism of this low mobility should be further investigated.

According to the extracted values from the SdH oscillation, the surface state conductance is determined as *G*_*s*_ = *n*_*s*_ *eμ*_*s*_ = 5.7 × 10^−4^ 1/Ω. This is consistent with the value *G*_*s*_ = 8.7 (e^2^/h) = 3.3 × 10^−4^ 1/Ω extracted from the thickness-dependent sheet conductance. This further supports that the thickness-dependent sheet conductance originates from the combination of the surface state and the bulk state.

**Figure 6:**
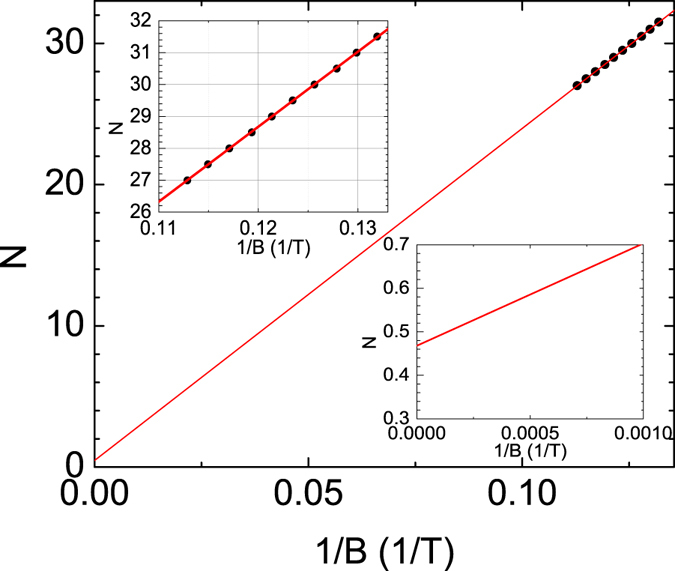
The Landau level fan diagram. The 0th Landau level is obtained from the linear extrapolation of 1/*B* = 0. The inset shows that the obtained value is 0.47 ± 0.01 which is consistent with the theoretical prediction for Dirac particles.

The Berry phase is a characteristic of the SdH oscillation. The Berry phase is 0 and *π* in the case of normal fermions with parabolic dispersion and Dirac fermions with a linear dispersion, respectively. One can extract the Berry phase value from the Landau level fan diagram^36^ which is indexed by the relation: 2*πN* = *F*/*B*_*N*_ + *φ*, where *N* is the *N*th Landau level, *F* is the SdH oscillation period, and *B*_*N*_ is the magnetic field of oscillation peaks. Figure 6 shows the Landau level fan diagram plot in 1/*B* versus the *N*th oscillation maxima and minima in the Δ*R*_*xx*_. The oscillation maxima and minima in Δ*R*_*xx*_ correspond to the integer value of *N* + 1/2 and *N*, respectively. To extract the Berry phase, a linear fitting of the Landau level fan diagram in Fig. 6 is done, which reveals that the intercept on the *N* axis is 0.47 ± 0.01. This result indicates that the Berry phase is *π*, which is consistent with the theoretical expectation for Dirac particles in the surface state of topological insulators.

## Conclusion

The electrical transport characteristic was investigated in a series of Sb_2_SeTe_2_ topological insulator nanosheets with the thickness ranging from 80 to 200 nm. The conductance increases as the thickness decreases, and the sheet conductance is proportional to the thickness. The corresponding sheet conductance of the surface state is 8.7 e^2^/h. The SdH oscillation was observed at hight magnetic fields and low temperatures. The oscillation frequency is 205 T that is corresponding to the *k*_*F*_ = 7.8 × 10^6^ cm^−1^. This extracted Fermi momentum is the same as the results from the value of ARPES, and the Berry phase is *π*. Following the L-K theory, the transport characteristics of the surface state is qualitatively determined. The surface state conductance is consistent with the determined value from the thickness-dependence sheet conductance. These support that the thickness-dependent sheet conductance originates from the combination of the surface state and the bulk state.
